# Comprehensive analysis of CXCL12 expression reveals the significance of inflammatory fibroblasts in bladder cancer carcinogenesis and progression

**DOI:** 10.1186/s12935-021-02314-y

**Published:** 2021-11-20

**Authors:** YiHeng Du, Jin Cao, Xiang Jiang, XiaoWei Cai, Bo Wang, Yi Wang, XiZhi Wang, BoXin Xue

**Affiliations:** 1 Department of Urology, The Second Affiliated Hospital of Soochow University, Suzhou, 215004 China; 2 Department of Urology, Suzhou Kowloon Hospital, Shanghai Jiaotong University School of Medicine, Suzhou, 215028 China; 3 Department of Pathology, Suzhou Kowloon Hospital, Shanghai Jiaotong University School of Medicine, Suzhou, 215028 China

**Keywords:** Bladder cancer, CXCL12, Cancer-associated fibroblasts, Carcinogenesis, Immunotherapy

## Abstract

**Background:**

Bladder cancer (BLCA) is the most common genitourinary tumor but lacks specific diagnostic biomarkers. Recent years have witnessed significant advances in the use and approval of immune checkpoint blockade (ICB) therapy to manage BLCA at advanced stages when platinum-based therapy has failed. The tumor microenvironment (TME) is essential in impacting BLCA patients' prognosis and responsiveness to ICB therapy. CXCL12 is a stromal secreted factor that was essentially involved in regulating the TME among cancers. In this article, we thoroughly investigated the TME regulating roles of CXCL12 in BLCA and revealed its critical involvement in the development of BLCA, which was closely correlated with inflammatory fibroblasts (iCAFs).

**Methods:**

We examined the gene expression profiles in the TCGA and GEO database to reveal the potential association of CXCL12 with the carcinogenesis and prognosis of BLCA. The receiver operating characteristic curve was used to explore the accuracy of CXCL12 along with multiple iCAFs-associated genes in the diagnosis of BLCA. The MCP-COUNTER, ESTIMATE, and TIDE algorithms were applied to estimate the TME components and predict immunotherapy responsiveness. An iCAFs signature was constructed using the ssGSEA algorithm. The "maftool" R package analyzed the oncogenic mutations in BLCA patients. Bioinformatics analysis results were further validated through immunohistochemistry of clinical samples. IMvigor210 cohort was used to validate bioinformatic predictions of therapeutic responsiveness to immune checkpoint inhibitors.

**Results:**

This manuscript revealed a significantly reduced expression of CXCL12 in BLCA compared with normal tissue. The expressions of various marker genes for iCAFs were also reduced considerably in BLCA tissues, highlighting the reduction of iCAFs in the pathogenesis of BLCA. Further studies revealed that CXCL12 and iCAFs were associated with pathological features, TME remodeling and aging in BLCA patients. The iCAFs signature further confirmed the intricate immunomodulatory roles of iCAFs in BLCA. Gene mutation analysis revealed the essential relationship between iCAFs and the mutation frequency of oncogenic genes, including TP53 and FGFR3. Meantimes, iCAFs levels also significantly affected BLCA patients' mutations in the TP53 and RTK-RAS pathways. Finally, our results confirmed the significant exclusion of CD8 + T cells by iCAFs, which further influenced the immunotherapy responsiveness in BLCA patients.

**Conclusions:**

This article highlighted the impact of CXCL12 on the pathogenesis and progression of BLCA. The reduced expression levels of iCAFs markers, including CXCL12, were highly accurate in the diagnosis of BLCA, suggesting the reduction of iCAFs accompanied bladder carcinogenesis. However, both CXCL12 and iCAFs significantly impacted the prognosis and immunotherapy responsiveness for BLCA patients by remodeling the TME. Our results critically suggested the dual roles of iCAFs in the carcinogenesis and progression of BLCA. Further exploration of iCAFs might unravel potential diagnostic biomarkers and therapeutic targets for BLCA.

## Introduction

Bladder cancer (BLCA) is a highly prevalent disease ranked eleventh among the most common causes of cancer-related deaths worldwide [1]. The absence of specific diagnostic markers for BLCA has caused many patients to miss the prime time for treatment. In most cases, patients diagnosed with BLCA initially present with non-muscle invasive BLCA (NMIBC), managed through transurethral resection of bladder tumor (TURBT) with or without intravesical treatments, including Bacille Calmette-Guerin (BCG) immunotherapy [2]. Although advancements in surgical techniques and multimodal therapy exist, the 5-year survival rates for muscle-invasive bladder cancer (MIBC) patients remain relatively low [3]. Recent evidence indicates that several immune therapy strategies in human cancers, including immune checkpoint blockade (ICB), show good clinical activity [4]. However, their effectiveness differ among patients.

The tumor microenvironment (TME) comprises immune and stromal components characterized by hypoxia, low extracellular pH, and high interstitial fluid pressure [5]. Studies show that TME exhibits pronounced heterogeneity and is suggested to promote tumor growth [6]. Recent evidence demonstrated a significant role for cancer-associated fibroblasts (CAFs) in the complex interaction between TME and tumor cells [7]. Considerable research has revealed that CAFs play a pivotal role in TME remodeling, influencing patients' responsiveness to ICB therapy [8]. There is evidence that CAFs critically support tumor progression, chemoresistance, metastasis [9], maintain cancer stem cells by producing growth factors, chemokines, and extracellular matrix (ECM) [10]. CAFs secrete chemokines, including CXCL12, are essential in recruiting CD8 + T cells into the TME [11]. Studies have demonstrated that CAFs can attract and sequester CD8 + T cells in the extramural compartment [12], which is suggested to impair the effectiveness of ICB therapy.

With the rapid advancement in single-cell RNA sequencing, CAFs in solid human tumors are now classified into two subgroups, including myofibroblasts (myCAFs) and inflammatory fibroblasts (iCAFs) [13]. These two CAFs subtypes have been described in disparate locations relative to the cancer cells. MyCAFs are primarily located adjacent to cancer cells, whereas iCAFs are situated in the desmoplastic areas of the tumor, farther away from the cancer cells. At the same time, it has recently been found that the effect of fibroblasts on the TME is also related to senescence, with senescent fibroblasts exhibiting a senescence-associated secretory phenotype (SASP). Typical fibroblasts with SASP secreted 75 factors, including IL6, IL8, and IL10, showing a substantial similarity to iCAFs.

Recent studies demonstrated the prognosis-impacting function of CXCL12 and iCAFs [14]. However, the roles of CXCL12 and iCAFs in the carcinogenesis and the aging microenvironment [15] of BLCA were rarely discussed. As such, we further explored the roles of CXCL12 and iCAFs in BLCA. Our results revealed the dual regulating roles of CXCL12 in the carcinogenesis and progression of BLCA, which was closely associated with iCAFs. Further studies on iCAFs will deepen our understanding of BLCA development and uncover promising diagnostic biomarkers and therapeutic targets for BLCA.

## Methods and materials

### Raw data acquisition

Data of transcriptome profiling and the corresponding clinical information were retrieved from the TCGA database (https://portal.gdc.cancer.gov/), including 408 patients. The method of data acquisition and application complied with the guidelines and policies of the TCGA database. Then the RNAseq data were transformed into TPM. The validation cohort (GES13507) was downloaded from the Gene expression Omnibus (https://www.ncbi.nlm.nih.gov/geo/), including 165 primary BLCA patients and their corresponding clinical data. The IMvigor210 cohort was used to validate the relationship between iCAFs and ICB responsiveness [16].

### Receiver operating characteristic (ROC) analysis

The ROC curve was used to determine the accuracy of the iCAFs-associated gene for BLCA diagnosis. A larger area under the curve (AUC) indicated the higher diagnostic accuracy of the gene for BLCA.

### Survival analysis

Differences in the survival of patients were compared via the Kaplan–Meier (KM) survival analysis with the Log-rank test. Using the Log-rank test, we calculated the P-value and hazard ratio (HR) with a 95% confidence interval (CI). The R package "survival" and "survminer "was applied to plot the KM curves.

### Screening the differentially expressed genes (DEGs) and functional analysis

The Limma package of R software was applied to evaluate the DEGs between the normal and tumor samples and the CXCL12 high and low expression group. DEGs were defined as genes with adjusted P < 0.05 and |Log2 (Fold Change)|> 1. Gene Ontology (GO)analysis on molecular function (MF), biological pathways (BP), and cellular components (CC) was performed for functional annotation. An analytical study of the DEGs' functions was achieved via Kyoto Encyclopedia of Genes and Genomes (KEGG) Enrichment Analysis. The 'ClusterProfiler' package of R software was applied for GO function analysis of potential targets and KEGG pathway enrichment analysis. For Gene sets enrichment analysis (GSEA), we employed the GSEA program acquired from the Broad Institute (http://www.broadinstitute.org/gsea/index.jsp). The Hallmark v7.2, c2 Kegg, and c5 Go (BP, CC, MF) gene sets were used for GSEA analysis.

### Estimating stromal cells and tumor infiltrated immune cells (TIICs)

The abundance of both the immune and stromal components, including CD8 + T cells, macrophages, endothelial cells and CAFs, was assessed using the MCP-COUNTER [17] algorithm from the 'immunedeconv' [18] R software package. The R package 'estimate' was employed to evaluate TME components and tumor purity scores. Subsequently, scores of stromal and immune components were acquired.

### Evaluating T cell exclusion and dysfunction and predicting ICB treatment reactiveness

The TIDE algorithm was used to predict potential ICB response [19]. TIDE integrates various gene expression markers to analyze two distinct mechanisms of tumor immune escape, tumor-infiltrating cytotoxic T lymphocyte (CTL) dysfunction and CTL exclusion, by immunosuppressive factors. A higher TIDE score denotes poorer efficacy of immune checkpoint blocking therapy (ICB).

### Single-sample gene set enrichment analysis (ssGSEA)

The abundance of iCAFs was explored via the ssGSEA [20] in the R Bioconductor package Gene Set Variation Analysis (GSVA, v.3.5). The ssGSEA algorithm is a rank-based method defining a score representing the degree of absolute enrichment of a particular gene set in each sample. We calculated the iCAFs abundance using iCAFs markers, including PDGFRA, CXCL12, CFD, DPT, LMNA, AGTR1, HAS1, CXCL1, CXCL2, CCL2, IL6, and IL8 [21].

### Gene mutation analysis

The somatic mutation information was retrieved from the TCGA database and visualized using the R package "maftools." the waterfall plot showed mutation data of each gene. Specific mutation types were annotated by different colors at the bottom left of the waterfall plot. The alteration of oncogenic pathways was also analyzed regarding the iCAFs levels.

### BLCA molecular subtype and single-cell data acquisition

The molecular subtype of BLCA was retrieved from two previously published articles, which classified BLCA into five [22] and six [23] subtypes. The TISCH database was used for detecting the stromal expressions of CXCL12 and PDGFRA at the single-cell level in BLCA.

### Sample collection and IHC analysis

Thirty post-operative BLCA sections from 2017–2020 were recruited for IHC analysis with the approval of the institutional ethics committee. Patients' clinical information was listed in the following table (Table 1). CXCL12 (Cell signaling technology, catalog number: 97958, 1:200), PDGFRA (Abcam, catalog number: ab134123, 1:500), VIM (Abcam, catalog number: ab92547, 1:500) and CD8A (Abcam, catalog number: ab237709, 1:100) expressions in BLCA sections were detected using the Benchmark GX automatic multifunctional immunohistochemical staining system (Roche, Switzerland) with Opti View DAB Detection Kit (Ventana, USA) following the manufacturer's protocol. A horseradish peroxidase-labeled secondary antibody visualized the primary antibodies. Hematoxylin was applied for counterstaining and Bluing Reagent for post counterstaining. We examined the expression of CXCL12, PDGFRA and CD8A in 3 consecutive slices in each section. Three most typical areas were selected to discuss the association of stromal and intra-tumor CD8 + T cell infiltration with the expression of CXCL12 and PDGFRA. ImageJ (version 1.50) was used to measure the stromal and intra-tumoral area and T cell counts. The adjacent normal tissues were used for the detection of CXCL12, PDGFRA and VIM.

**Table 1:** Clinical information for BLCA patients with IHC analysis

Characteristics	Age		Gender		T stage		N stage		M stage		Grade	
	≤ 65	> 65	Male	Female	Ta–T1	T2–T4	N–	N+	M−	M+	High	Low
Number	14	16	20	10	19	11	29	1	28	2	25	5

### Statistics analysis

The univariate logistics regression was used for the association of CXCL12 with BCLA pathological features. The Wilcoxon test examined the differences between variables of two groups. Kruskal Wallis test analyzed statistical significance for variables of more than two groups. The Spearman analysis was used for the correlation test. The Chi-square test was used to identify the correlation of iCAFs with oncogenic mutations and ICB responsiveness. Two sides P-value < 0.05 was considered significant. R language v4.0.3 was used for all statistical analyses.

## Results

### Reduction of CXCL12 expression was potentially associated with BLCA carcinogenesis

**Fig. 1:**
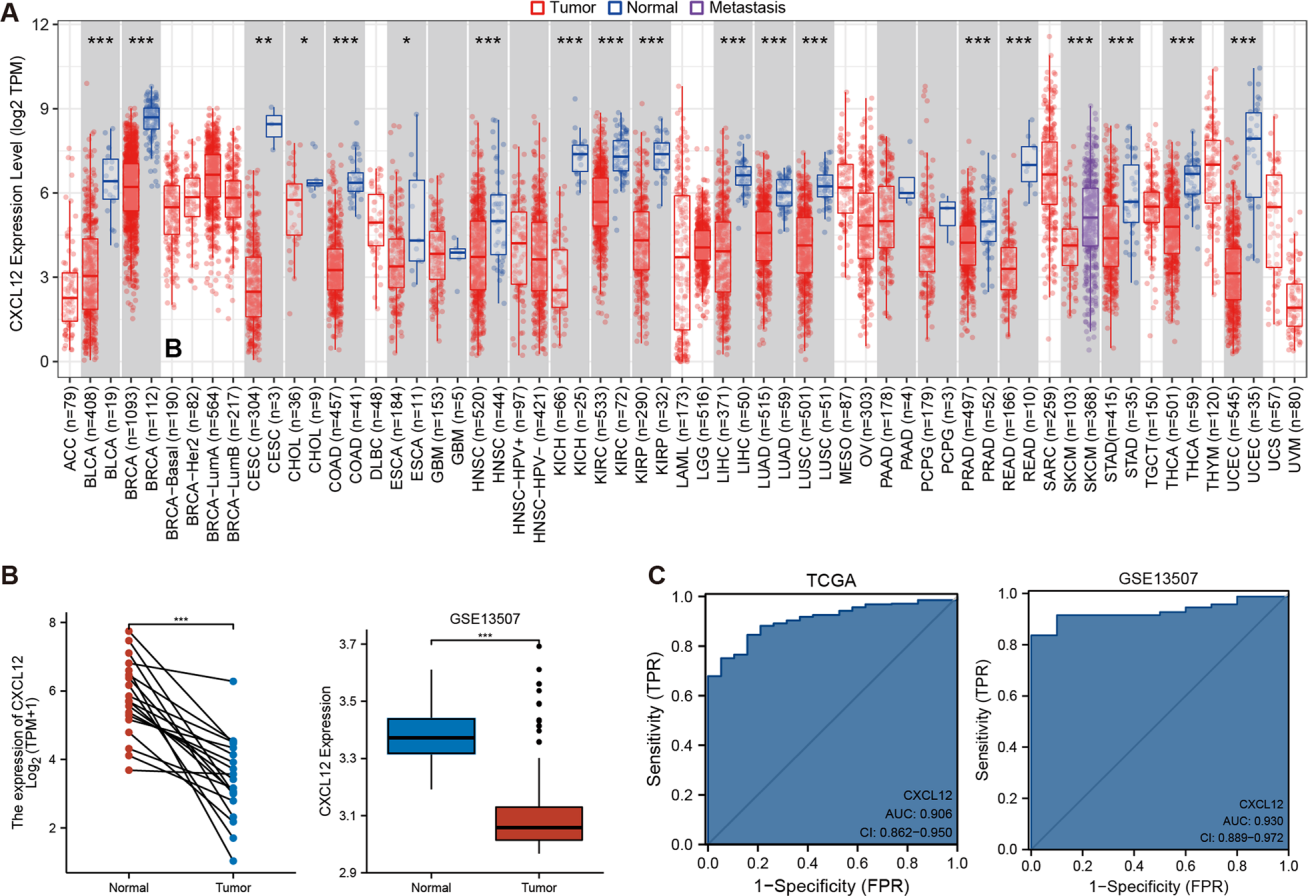
The reduction of CXCL12 expression was involved in the carcinogenesis of BLCA. **A**, **B** The expression of CXCL12 was significantly lower in bladder cancer compared with normal tissue in TCGA and GEO cohorts (p < 0.001). **C** ROC curves showed the high accuracy of CXCL12 in predicting bladder cancer pathogenesis. *** p < 0.001, **p < 0.01, *p < 0.05

CXCL12 expression was explored across tumor types in the TCGA database, followed by paired differential analysis. CXCL12 expression was further validated in the GEO dataset (GSE13507). Significantly lower expression of CXCL12 was reported in tumor tissues among various tumor types, including BLCA (p < 0.001) (Fig. 1A, B). Moreover, the contribution of decreased CXCL12 expression level in BLCA diagnosis was explored using the ROC curve. The results demonstrated that the decreased CXCL12 expression allowed for highly accurate BLCA diagnosis in both TCGA (AUC = 0.906) and GEO datasets (AUC = 0.930) (Fig. 1C). These data strongly suggest that CXCL12 is of significance in BLCA pathogenesis.

### The reduction of CXCL12 expression was closely associated with the decreased abundance of iCAFs in BLCA pathogenesis

**Fig. 2:**
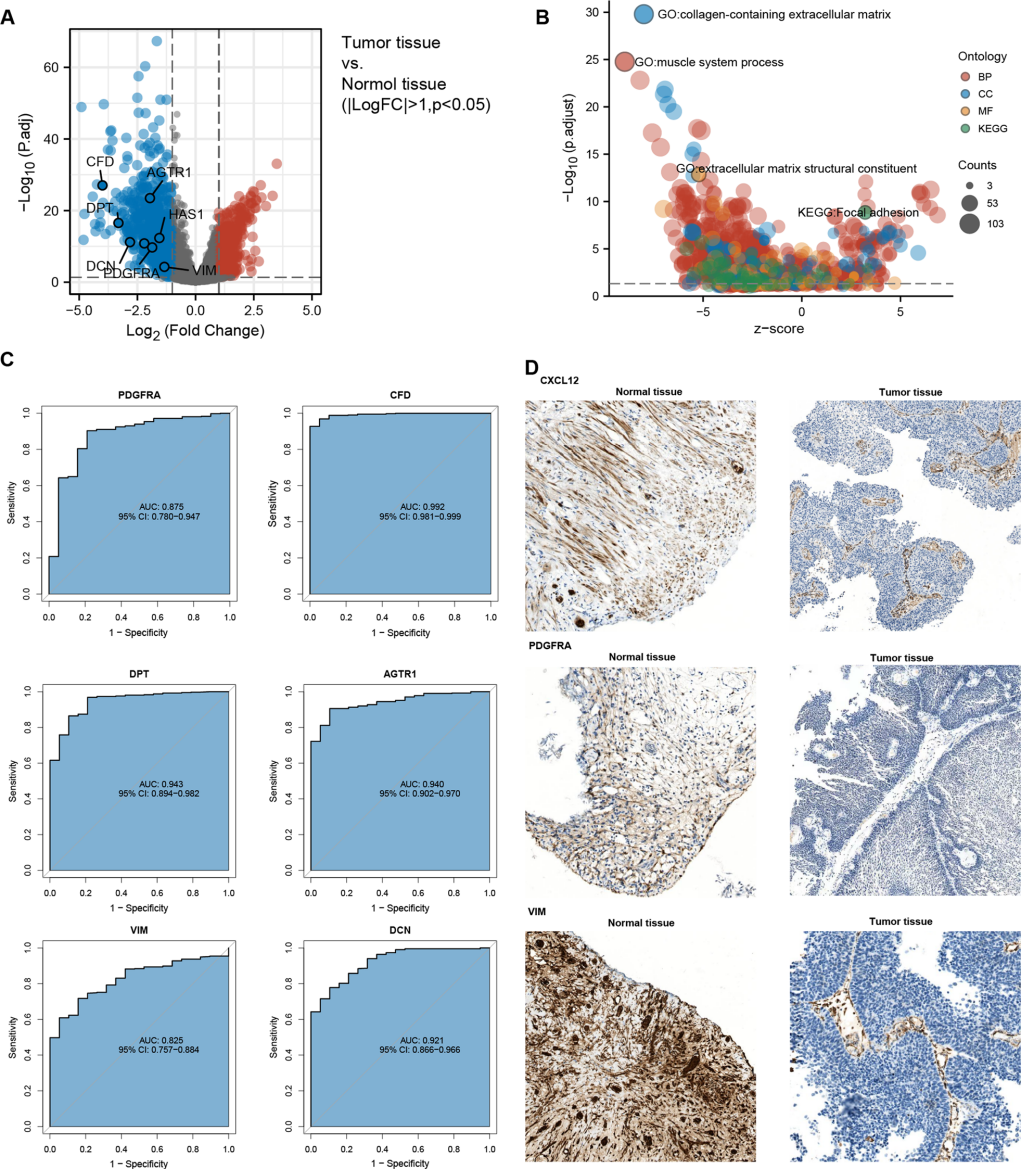
The decrease of iCAFs accompanied BLCA pathogenesis. **A** The expression of marker genes for iCAFs was significantly reduced in BLCA compared with normal tissues. **B** GO and KEGG enrichment analysis indicated decreased extracellular matrix in BLCA, further suggesting reduced fibroblasts in BLCA pathogenesis. **C** ROC curves showed high diagnostic values of various iCAFs marker genes. **D** Immunohistochemistry assays confirmed the reduction of iCAFs markers in BLCA, including CXCL12, PDGFRA and VIM

To further discuss the potential mechanisms of CXCL12 expression reduction in BLCA pathogenesis, we analyzed the DEGs between normal and bladder tumor tissues. Similar to CXCL12, multiple markers of iCAFs were significantly reduced in tumor tissues (Fig. 2A). Meantime, GO and KEGG enrichment analysis revealed that the collagen-containing extracellular matrix was decreased considerably, highlighting the reduction of CAFs within the process of BCLA carcinogenesis (Fig. 2B). We performed ROC curve analysis to further examine the diagnostic accuracy of reduced expression levels of iCAFs-related genes for BLCA. We surprisingly found that reduced expression levels of these genes were highly diagnostic for BCLA, including PDGFRA (AUC = 0.875), CFD (AUC = 0.992), DPT (AUC = 0.943), AGTR1 (AUC = 0.940), VIM (AUC = 0.825), and DCN (AUC = 0.921) (Fig. 2C). Immunohistological assays were applied to validate the expression of CXCL12, PDGFRA and VIM in normal and tumor tissues (Fig. 2D). The results corresponded with the RNA sequencing results gained from the TCGA database, showing significant reductions of these genes in early-stage BLCA sections. All these results emphasized the reduction of iCAFs within the process of BLCA pathogenesis.

### The CXCL12 expression level was significantly associated with BLCA prognosis and clinical features

**Fig. 3:**
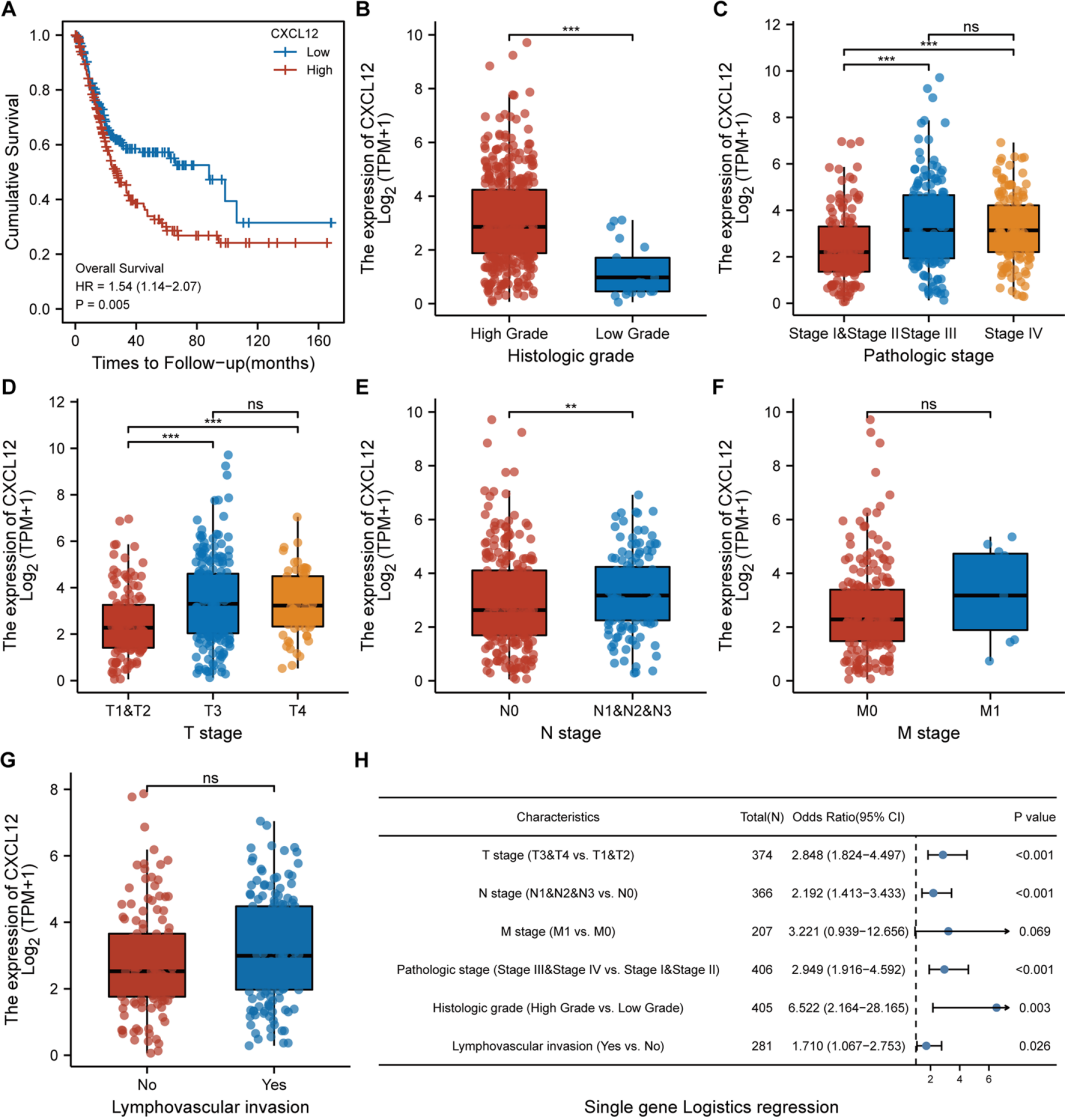
The impact of CXCL12 on BLCA survival and clinical features. **A** Patients with high CXCL12 expression tended to have a relatively lower OS (p = 0.005). **B**–**G** CXCL12 expression was upregulated in patients with relatively advanced bladder cancers. **H** The single-gene logistics regression indicated that CXCL12 expression is correlated with BLCA pathological features, including grade (p = 0.003), stage (p < 0.001), T classification (p < 0.001), N classification (p < 0.001)and lymphovascular invasion (p = 0.026). *** p < 0.001, **p < 0.01. *ns* not significant

The influence of CXCL12 expression levels on the prognosis and clinical characteristics of BLCA was explored by analyzing the difference in OS between the CXCL12^high^ and CXCL12^low^ groups. The OS of patients in the CXCL12^high^ expression group was significantly lower than that of the CXCL12^low^ group (p = 0.005), suggesting that high CXCL12 expression was an unfavorable factor for BLCA prognosis (Fig. 3A). Further exploration of the CXCL12 expression levels between patients with different clinical statuses demonstrated significantly higher CXCL12 expression in BLCA patients with higher tumor grade (p < 0.001), advanced clinical stage (p < 0.001), lymph node metastasis (p < 0.01) (Fig. 3B–G). Single-gene logistics regression analysis supported the view that CXCL12 expression level was a detrimental indicator of the clinicopathological features of BLCA (Fig. 3H).

### CXCL12 played multiple regulatory functions in the tumor microenvironment

**Fig. 4:**
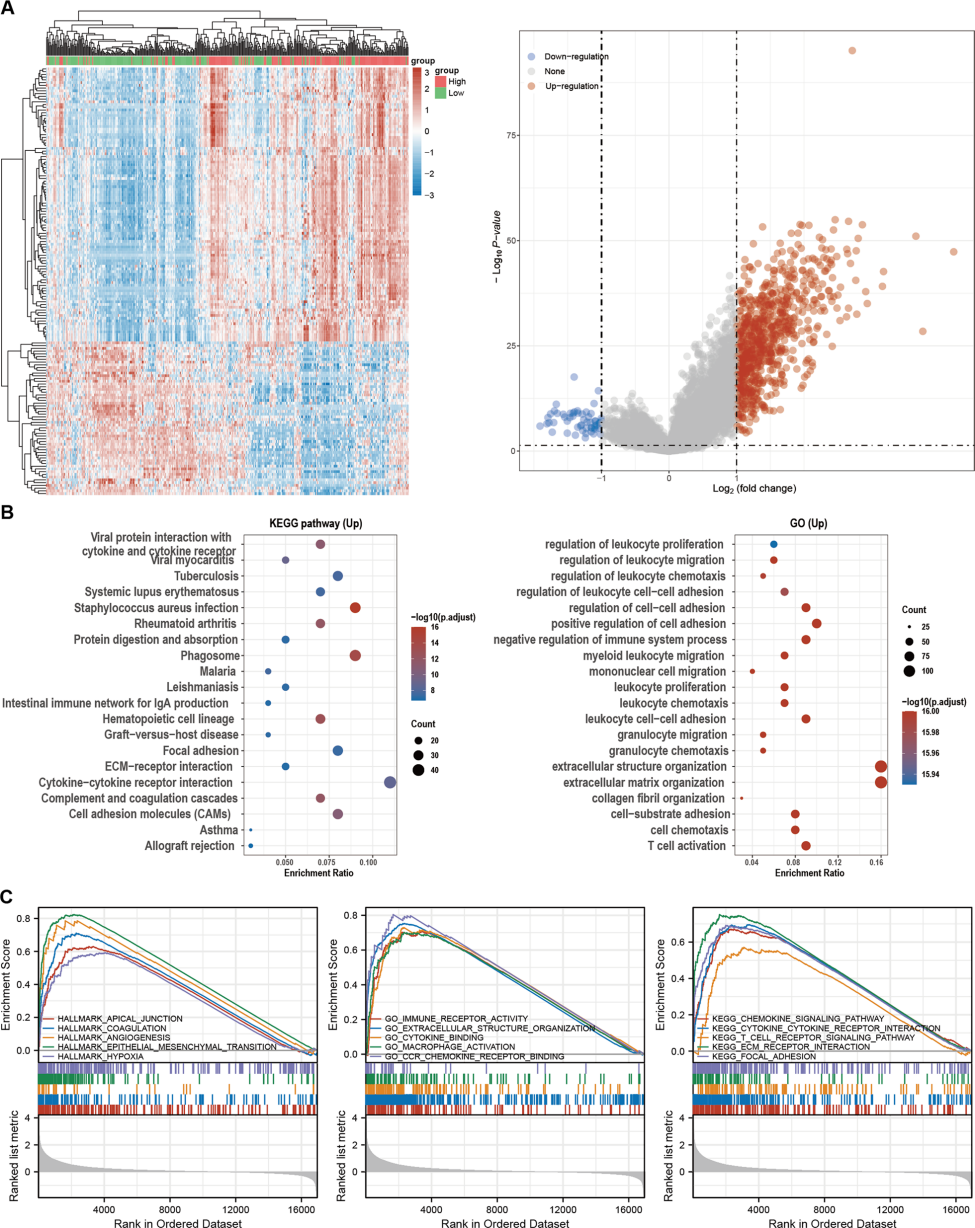
Functional annotation of CXCL12 by GO, KEGG and GSEA. **A** The heatmap showed the top 50 upregulated and downregulated DEGs between high- and low-CXCL12 expression groups, and the volcano plot showed the upregulated and downregulated DEGs. **B** KEGG and GO enrichment analyses showed that CXCL12 is related to multiple ECM remodeling and immune-related processes. **C** GSEA analyses revealed that CXCL12 remodels the stromal and immune components

Analysis of the DEGs between CXCL12^high^ and CXCL12^low^ groups in the TCGA BLCA cohort revealed 757 upregulated and 56 down-regulated DEGs (Fig. 4A). GO and KEGG enrichment analysis revealed the significant involvement of CXCL12 in KEGG pathways, including Cytokine-cytokine interaction, cell adhesion cams and ECM-receptor interaction, and GO functions, including extracellular structure organization and extracellular matrix organization (Fig. 4B). GSEA suggested high enrichment of CXCL12 in hallmark gene sets, including epithelial to mesenchymal transition, angiogenesis, and hypoxia. While it was also indicated that CXCL12 was essentially involved in GO gene sets encompassing immune receptor activity, cytokine binding, chemokine receptor binding, and macrophage activation. Lastly, the KEGG gene sets demonstrated that CXCL12 was associated with pathways covering chemokine and T cell receptor signaling (Fig. 4C). These results indicated an essential role for CXCL12 in TME remodeling.

### CXCL12 was correlated with multiple immune checkpoint-related genes, TIICs, stromal cells and the aging microenvironment.

**Fig. 5:**
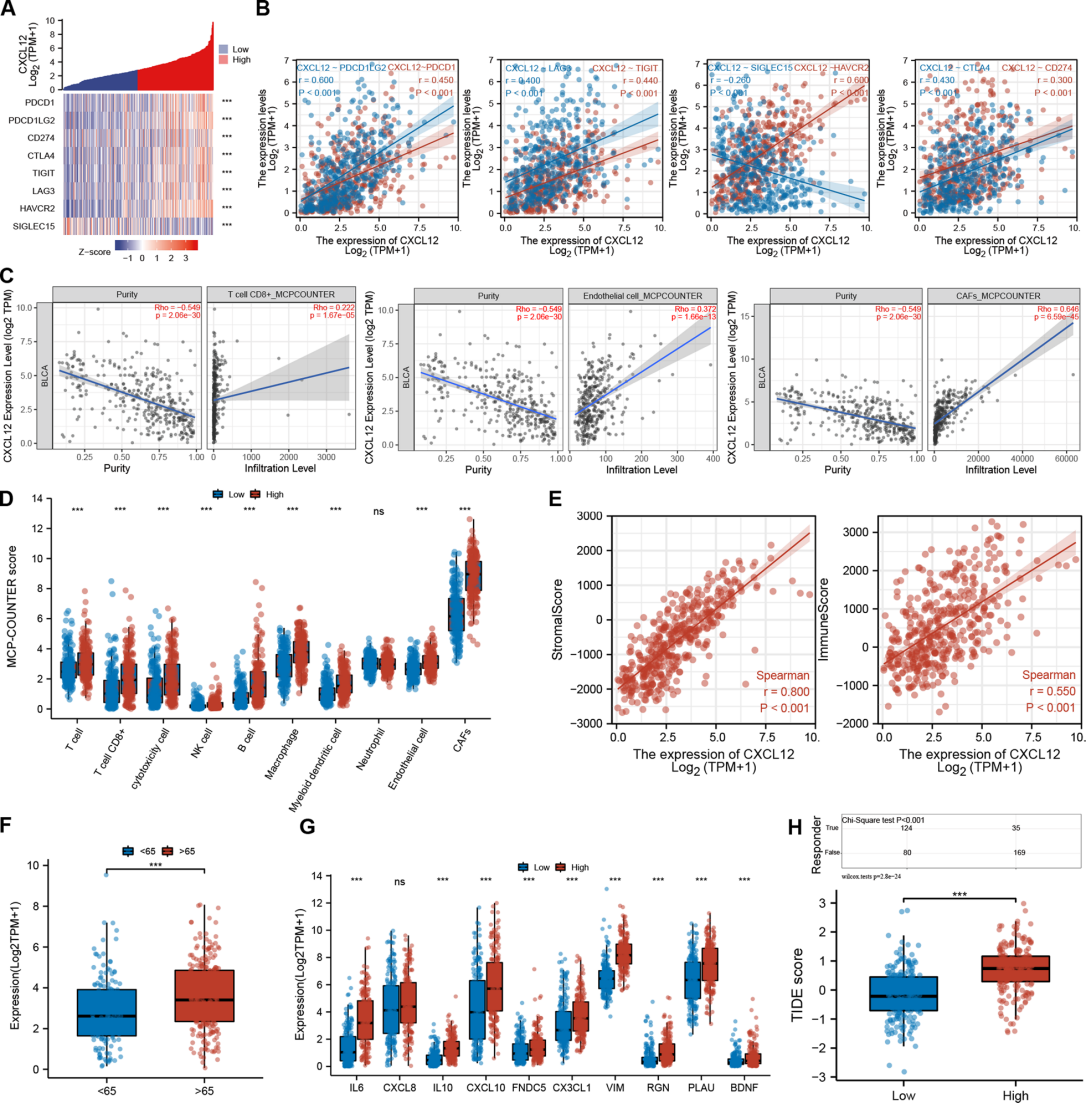
CXCL12 correlated with the TME of BLCA that influenced patients' responsiveness to ICB therapy. **A**, **B** CXCL12 is positively correlated with immune checkpoint genes, including PDCD1, PDCD1LG2, CD274, CTLA4, TIGIT, LAG3, and HAVCR2, but negatively related to SIGLEC15. **C**, **D** The MCP-COUNTER algorithm indicated that CXCL12 expression level is positively associated with CD8 + T cells, endothelial cells and CAFs in the TME. **E** The ESTIMATE algorithm showed a positive correlation of CXCL12 expression with the stromal and immune scores. **F**, **G** CXCL12 was associated with patients' age, SASP and aging-related genes. **H** The TIDE algorithm demonstrated that CXCL12 expression influenced the responsiveness of BLCA patients to ICB therapy. *** p < 0.001, ns: not significant

We further analyzed the association between CXCL12 and eight immune checkpoint-related genes, including PDCD1, PDCD1LG2, CD274, CTLA4, TIGIT, LAG3, HAVCR2, and SIGLEC15. Results demonstrated that CXCL12 expression was positively correlated with PDCD1 (R = 0.450, p < 0.001), PDCD1LG2 (R = 0.600, p < 0.001), CD274 (R = 0.300, p < 0.001), CTLA4 (R = 0.430, p < 0.001), TIGIT (R = 0.440, p < 0.001), LAG3 (R = 0.400, p < 0.001), and HAVCR2 (R = 0.600, p < 0.001) and negatively correlated with SIGLEC15 (R = -0.260, p < 0.001) (Fig. 5A, B). Subsequently, using the MCP-COUNTER algorithm, we explored the abundance of immune and stromal cells, including CD8 + T cells, endothelial cells and CAFs. The Spearman correlation test showed a positive correlation of CXCL12 with CD8 + T cells (R = 0.222, p < 0.001), endothelial cells (R = 0.372, p < 0.001) and CAFs (R = 0.646, p < 0.001) (Fig. 5C, D). The ESTIMATE algorithm disclosed a profound association of CXCL12 with both the stromal score (R = 0.800, p < 0.001) and the immune score (R = 0.550, p < 0.001) (Fig. 5E). Recent studies have emphasized the significance of aging in influencing the TME. Therefore, we further analyzed the levels of CXCL12 between patients with age below 65 and above 65. Our data indicated significantly higher CXCL12 expression in older patients (Fig. 5F), and further analysis revealed a significant positive association of CXCL12 with SASP and multiple age-related genes[22] (Fig. 5G). These results highlight the influence of the aging microenvironment on CXCL12 expression, which could further promote tumor progression. As an iCAFs secreted protein, CXCL12 potentially attracts T cells to the stromal area, contributing to cell exclusion and dysfunction. In this view, the TIDE algorithm was employed to evaluate the T cell dysfunction and exclusion level. Results revealed that CXCL12 was significantly positively correlated with the TIDE score, suggesting a substantial role for CXCL12 in T cell depletion in the tumor immune response. Subsequently, the responses of patients with high and low CXCL12 expression levels to ICB treatment were also evaluated. Correspondingly, patients expressing high CXCL12 levels tended to exhibit impaired responses to ICB therapy (p < 0.001) (Fig. 5H). These findings provide evidence of the significant contribution of CXCL12 in the interaction of stromal with immune components. Such interaction causes immune suppression in the TME, impacting the patient's response to ICB therapy.

### Construction of ssGSEA-based signature for iCAFs

**Fig. 6:**
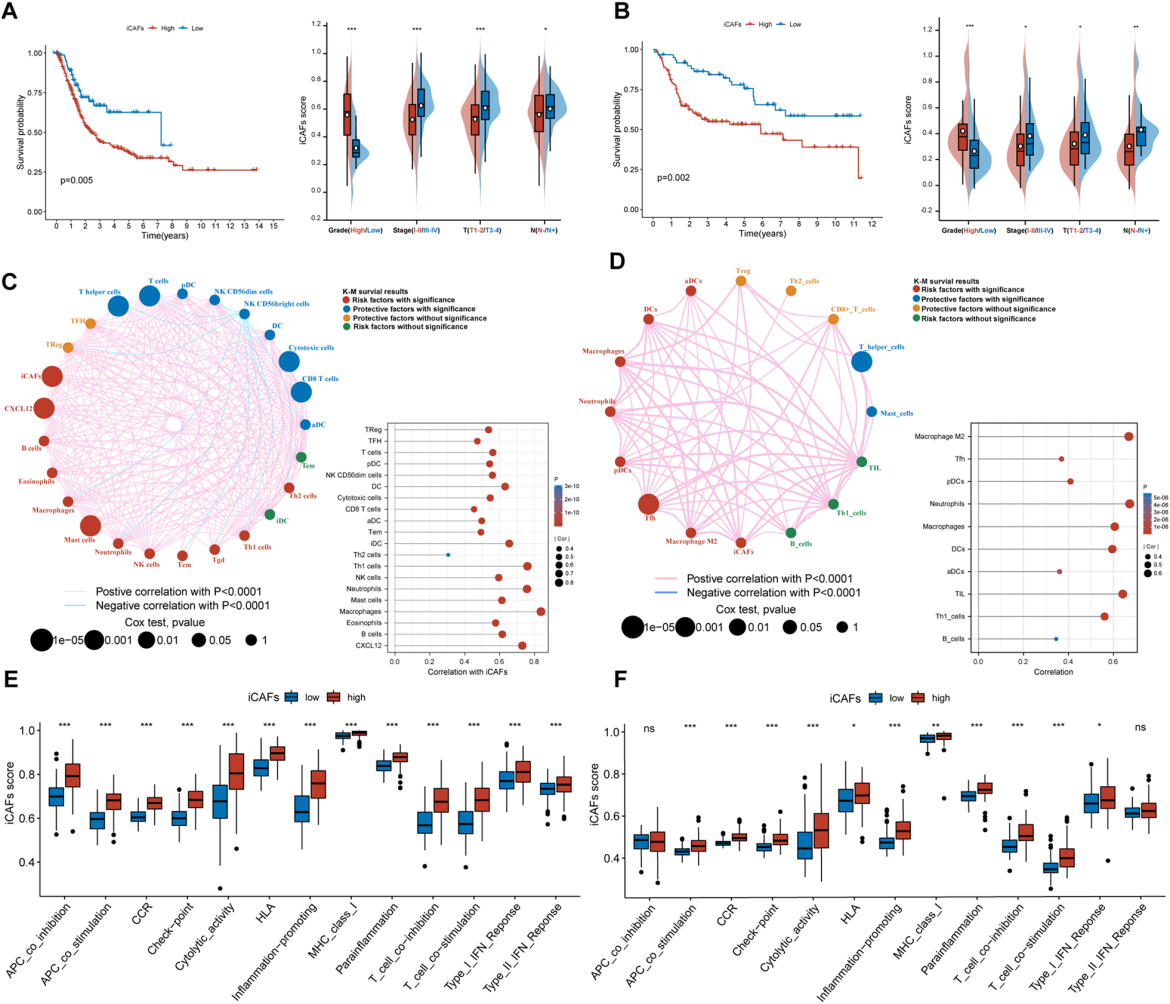
iCAFs score predicted poor prognosis of BLCA patients. **A**, **B** iCAFs score was associated with poor OS of patients with BLCA in the TCGA and GEO cohorts, with higher iCAFs scores recorded in advanced BLCA. **C**, **D** iCAFs scores were positively associated with the expression of CXCL12, T cells and macrophage infiltration. **E**, **F** iCAFs were involved in multiple immune-related functions, including CCR, checkpoint, inflammation-promoting and T cell regulation. *** p < 0.001, **p < 0.01, *p < 0.05. *ns* not significant

The effect of iCAFs on BLCA was investigated using the iCAFs signature, which was constructed by the ssGSEA algorithm based on the markers of iCAFs. Subsequently, we explored the relationship of iCAFs with the prognosis and clinical features of TCGA BLCA patients. The OS of patients with high iCAFs scores was significantly lower than that in patients with low iCAFs scores (p = 0.005). Meanwhile, in different clinical stages, the iCAFs score of patients increased with tumor up-staging (Fig. 6A). Furthermore, the prognostic role of the iCAFs score (p = 0.002) was validated in GEO datasets (Fig. 6B). The correlation analysis provided more evidence on the significant association of iCAFs score with CXCL12, T cells and macrophages (Fig. 6C, D). Using the ssGSEA algorithm, we further identified significant involvement of iCAFs in multiple immune-related functions, covering CCR, checkpoint, inflammation-promoting and T cell regulation (Fig. 6E, F).

### iCAFs score is correlated with oncogenic mutations, molecular subtype, and immune therapy responsiveness.

**Fig. 7:**
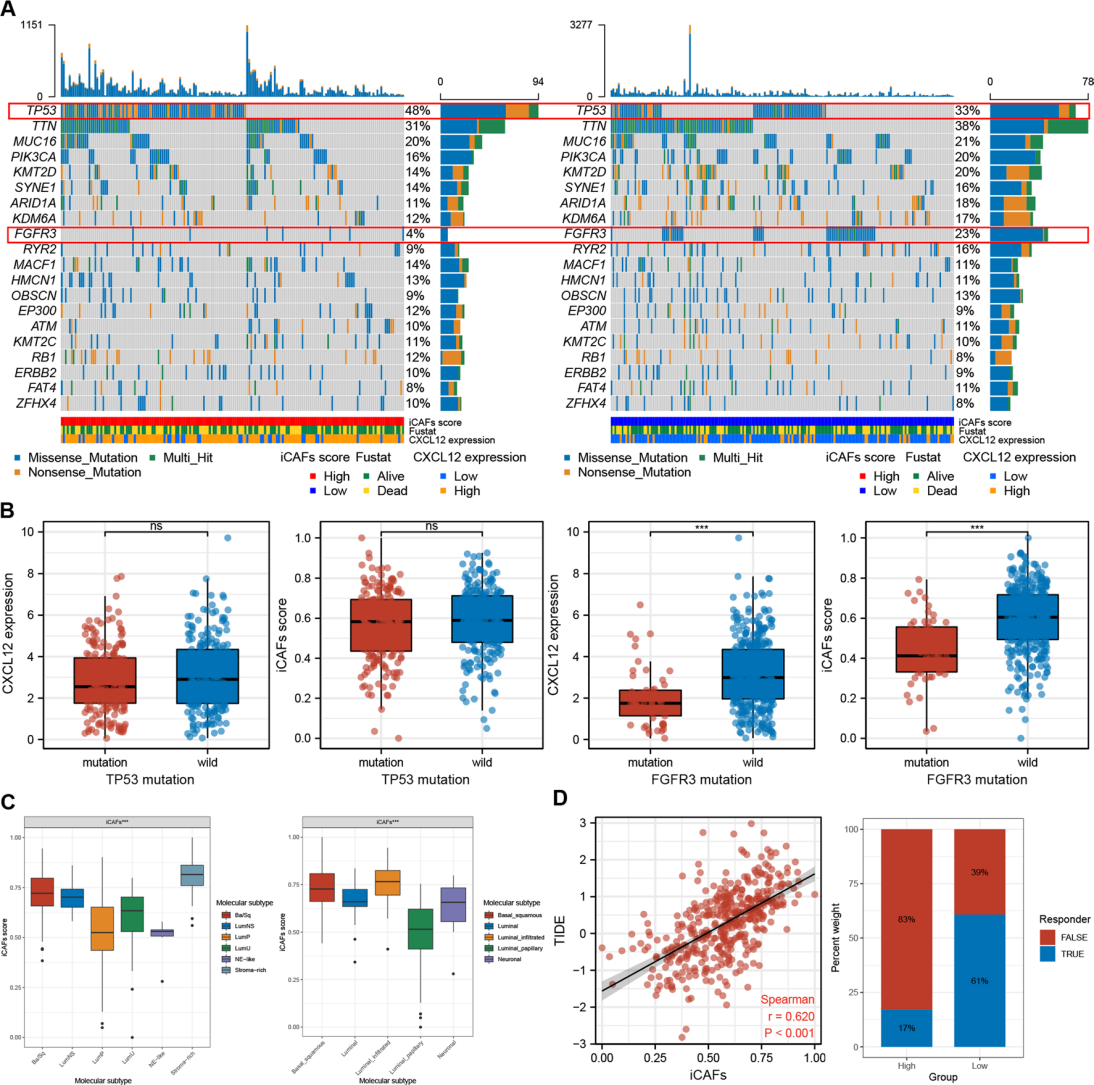
The correlations of iCAFs scores with gene mutation, molecular subtype and ICB responsiveness. **A**, **B** The iCAFs score was negatively correlated with the mutation frequency of FGFR3. **C** iCAFs score was significantly upregulated in BLCA with stromal rich molecular subtype. **D** The TIDE algorithm indicated significantly lower responsiveness in high iCAFs patients, possibly due to the dysfunction and exclusion of T cells. *** p < 0.001, ns: not significant

Correlation analysis between iCAFs scores and gene mutations revealed a significantly higher frequency of TP53 mutations (p = 0.005)and markedly lower frequency of FGFR3 mutations (p < 0.001) in the iCAFs^high^ group (Fig. 7A) (Table 2). Patients in the TP53 mutation and the wild-type groups exhibited no significant difference in CXCL12 expression and iCAFs scores. Contrarily, significantly lower CXCL12 levels and iCAFs scores were reported in patients in the FGFR3 mutation group than those in the FGFR3 wild-type group (Fig. 7B). The analysis on the alteration of oncogenic pathways indicated a significant influence of iCAFs levels on the RTK-RAS and TP53 signaling pathways (Table 2). Comparison of the iCAFs scores between patients with different molecular subtypes revealed high concordance between the iCAFs scores and the characteristics of each subtype. Notably, the iCAFs scores were significantly higher in the fibroblast-rich molecular subtypes, such as stromal infiltrated, but markedly lower in the luminal papillary, which earned the least stromal components (Fig. 7C). Correlation analysis between the TIDE and iCAFs scores showed a strong correlation between iCAFs scores and T cell dysfunction and exclusion (R = 0.620, p < 0.001). By ICB responsiveness prediction of TIDE algorithm, we also found that iCAFs score significantly influenced ICB responsiveness BLCA patients (p < 0.001) (Fig. 7D).

**Table 2:** Difference of gene and pathway mutations with significance between iCAFs high and low groups of TCGA BLCA patients

Gene	iCAFs high-wild	iCAFs High-mutation	iCAFs Low-wild	iCAFs Low-mutation	p-value
FGFR3	190 (96.45%)	7 (3.55%)	158 (77.45%)	46 (22.55%)	< 0.001
TP53	103 (52.28%)	94 (47.72%)	136 (66.67%)	68 (33.33%)	0.005

Pathways	iCAFs high-affected	iCAFs high-not affected	iCAFs low-affected	iCAFs low-not affected	p-value
TP53	112 (56.85%)	85 (43.15%)	152 (74.51%)	52 (25.49%)	< 0.001
RTK-RAS	106 (53.81%)	91 (46.19%)	86 (42.16%)	118 (57.84%)	0.025

### CXCL12 was closely associated with iCAFs, CD8 + T cells exclusion, and clinical-pathological features

**Fig. 8:**
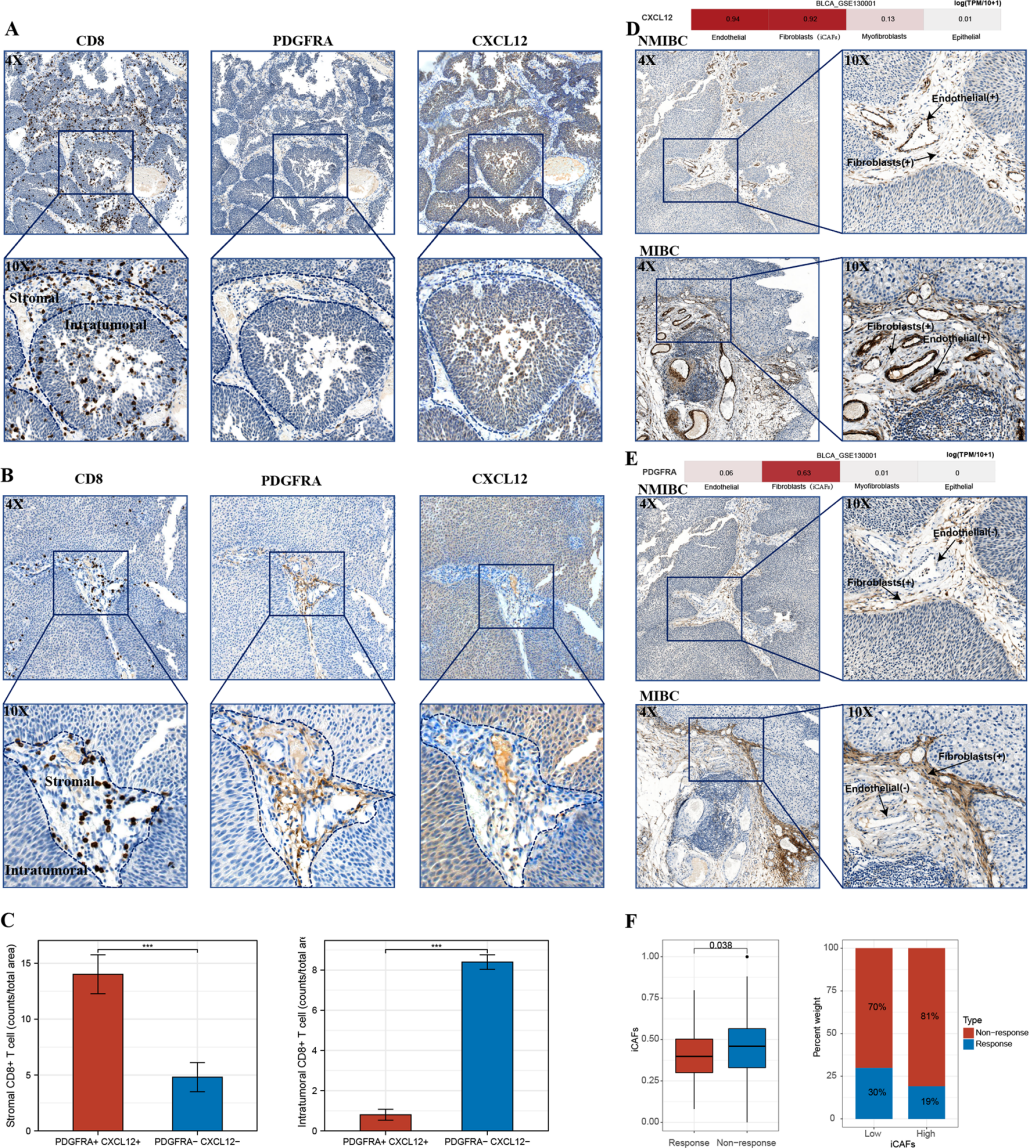
IHC verification of the association of CXCL12 and iCAFs with CD8 + cells infiltration. **A** Intratumoral CD8 + T cells were observed in tumors with negative stromal CXCL12 and PDGFRA expression. **B** Significant infiltration of CD8 + T cells was found in the stromal components of CXCL12 and PDGFRA positive tumors, highlighting the exclusion of CD8 + T cells by iCAFs. **C** Statistics analysis indicated a significant decrease of intratumoral CD8 T cells in iCAFs positive tumors. **D** CXCL12 expression was expressed by endothelial cells and iCAFs in BLCA and was upregulated in MIBC. **E** PDGFRA was mainly expressed by iCAFs in BLCA, upregulated in MIBC. **F** The IMvigor210 cohort confirmed the iCAFs score was correlated with patients' responsiveness to ICB therapy. *** p < 0.001

IHC assay demonstrated that tumors with significant CD8 + T-cell intra-tumoral infiltration usually exhibited negative CXCL12 and PDGFRA expression in the stromal components (Fig. 8A). Contrarily, stromal CXCL12 and PDGFRA positive tumors showed high CD8 + T-cell infiltration in the stromal compartment accompanied by low intra-tumoral infiltration (Fig. 8B). These results suggest that the expression of stromal PDGFRA and CXCL12, namely the presence of iCAFs, played an essential role in T-cell clearance. We further quantified and validated the CD8 + T cell were excluded and sequestered in the stromal compartments where iCAFs were located (Fig. 8C).

Subsequent examination of CXCL12 expression in BLCA of different invasive features suggested higher expressions of CXCL12 and more stromal components in MIBC compared with NMIBC. Meanwhile, we further observed the expression of CXCL12 by endothelial cells and iCAFs, while PDGFRA was dominantly expressed by iCAFs. These results coordinated with the single-cell RNA sequencing results gained from the TISCH database (Fig. 8D, E). More importantly, we confirmed that PDGFRA and CXCL12 are co-expressed by iCAFs in stromal components through these immunohistochemical sections. Finally, Sequencing results from the IMvigor210 cohort validate a significant association between ssGSEA-constructed iCAFs scores and patients' responsiveness to immunotherapy (p = 0.038) (Fig. 8F).

## Discussion

The prevalence and incidence of BLCA are seeing an unprecedented rise worldwide [23]. BLCA can be classified into NMIBC and MIBC based on tumor invasion depth. MIBC is a lethal type, which warrants definitive treatment. For many years, researchers are yet to make significant progress in the treatment of MIBC, especially after the failure of platinum-based chemotherapy. Recently, several clinical pieces of evidence have validated the remarkable effectiveness of immune checkpoint inhibitors in managing advanced BLCA. In this view, immune checkpoint inhibitors are now guideline-recommended therapy for advanced BLCA that has failed prior chemotherapy. Nevertheless, immune checkpoint inhibitors still face problems of low responsiveness and frequent adverse effects, which warrants urgent exploration of the strategies to increase the responsiveness of BLCA immunotherapy.

As the tumor cells begin to proliferate and invade, they initiate microenvironment remodeling by activating resident fibroblasts, which replace the adipocyte-rich stroma with CAFs [24]. The contribution of the microenvironment to tumor progression is underpinned by autocrine and paracrine signaling, in which the secretome of CAFs and cancer cells plays a pivotal role [25]. Recent advancement in single-cell RNA sequencing significantly enriches our understanding of the heterogeneity of the TME. There is a common view that the CAFs can be classified into subgroups, including iCAFs and myCAFs. The iCAFs, which earned profound secreting features and expressed PDGFRA and CXCL12, were confirmed to impact the prognosis of BLCA patients. Through single-cell RNA sequencing, iCAFs have been shown to exert essential functions in recruiting immune cells into the tumor microenvironment [14]. Recent evidence also supports that the direct interactions between CAFs and T cells, mediated via coincident upregulation and engagement of PD-1 on T cells, drive T cell dysfunction and death within tumors [26]. In this manuscript, we also observed the similar phenomena that iCAFs could essentially exclude CD8 + cells infiltrating into the tumor cells, highlighting the immune-modulating function of iCAFs in BLCA.

While the cancer-promoting and immune-modulating roles of iCAFs were well investigated in the previous literature, the significance of iCAFs in BLCA carcinogenesis was rarely discussed.

Our findings revealed that iCAFs were significantly reduced in early-stage BLCA compared with normal bladder tissue. Contrarily, iCAFs supported tumor progression and further impacted the overall survival of BLCA patients. These results gave the impression that iCAFs played a dual role in the pathogenesis and progression of BLCA. Previous studies have shown that fibroblasts were often observed circumscribing early or premalignant lesions, which supported the idea that the initial fibroblast response can be tumor suppressive [27, 28]. Meantimes, fibroblasts started to expand and generate protumorigenic ones with tumor progression, termed "stromagenesis." From this point of view, the fibroblasts reduced during the formation of BLCA likely played an anti-tumor role. During tumor formation, these anti-tumoral fibroblasts gradually perished and the tumor cells gradually became dominant, which eventually induced the production of pro-tumor fibroblasts, leading to tumor progression. However, this hypothesis still needs further verification. Nevertheless, along with the significant decrease of iCAFs during BLCA carcinogenesis, various marker genes of iCAFs, including CXCL12, CFD, DPT, and AGTR1, showed strong diagnostic value, which provided potential diagnostic biomarkers for BLCA.

Alternatively, our data also revealed that iCAFs owned a significant negative association with FGFR3 mutation frequency. Studies have confirmed that FGFR3 mutations in BLCA are often accompanied by FGFR3 over-expression(29), leading to the inhibition of CAFs formation, and these findings were consistent with our results. Meanwhile, our study also found a significant association between the abundance of iCAFs and mutations in TP53 and RTK-RAS signaling pathways, suggesting that an increased frequency of mutations accompanied the progression of BLCA in the TP53 and RTK-RAS signaling pathways.

Using bioinformatics methods such as GSEA, TIDE, ESTIMATE, and ssGSEA, we confirmed that CXCL12 is an unfavorable prognostic factor in the progression of BLCA. Underlying the impact of CXCL12 on the prognosis of BLCA patients existed a process of regulation of the TME by iCAFs. This process ultimately established an immunosuppressive and pro-tumor TME. Although previous studies have already confirmed these roles of CXCL12 in BLCA, we still chose CXCL12 as a representative of the many marker genes of iCAFs in this article to reveal aspects of iCAFs that were not well known in previous articles. We also found that the expression level of CXCL12 in the TME of BLCA was significantly correlated with the aging of BLCA patients and showed a significant correlation with SASP and multiple aging-related genes. The above results suggest that CXCL12's pro-tumor effects might also be associated with senescence, which is rarely reported in the previous literature. The in-depth study of CXCL12 and iCAFs may have particular implications for the senescent tumor microenvironment.

Finally, by exploring the expression of CXCL12, PDGFRA, and CD8A in BLCA specimens, it was demonstrated that PDGFRA and CXCL12 were co-expressed in iCAFs. Meanwhile, a significant increase of CD8 + T-cell infiltration was found in the stromal region where iCAFs existed. Intratumoral CD8 + T-cell infiltration was significantly elevated in tumor tissues negative for PDGFRA and CXCL12 expression. These findings mirrored the exclusive effect of iCAFs on CD8 + T cells through CXCL12. In addition, CXCL12 protein was found hyper-expressed in MIBCs, whereas only a small subset of NMIBC expressed CXCL12. Moreover, our immunohistochemical experiments demonstrated that CXCL12 could be represented by endothelial cells and fibroblasts in the stroma, while PDGFRA was mainly expressed by fibroblasts. The cells co-expressing CXCL12 and PDGFRA could indeed represent the iCAFs described in the literature. These results also laterally confirmed that the signature of iCAFs constructed by ssGSEA, which stood for the combined expression of iCAFs markers, could be used to represent the relative abundance of iCAFs. Finally, the IMvigor210 cohort further validated that the iCAFs may impact patients' responsiveness to ICB therapy. These findings affirmed the results of bioinformatics analysis, strongly suggesting that iCAFs played a part in the immunosuppression of BLCA and are potentially relevant factors in patients' responsiveness to ICB therapy.

Although the present study revealed remarkable findings, a few limitations cannot be ignored. First, our research is mainly based on bioinformatics analysis. In this view, experimental data are still needed to verify the specific roles of iCAFs and TME remodeling in BLCA. Secondly, the number of clinical samples analyzed was limited, and clinical cohorts with larger sample volumes are needed to verify the diagnostic potential of the iCAFs markers in BLCA carcinogenesis. Last, the mechanisms of iCAFs on impacting the BLCA patients' immunotherapy responsiveness still need further verification by in vivo and in vitro studies.

In conclusion, this work demonstrates the effect of CXCL12 on the occurrence and progression of BLCA at multiple levels through systematic multi-omics bioinformatics analysis along with immunohistochemical verification. Our results highlighted the dual functions of iCAFs on BLCA carcinogenesis and progression. The treatment targeting iCAFs may provide new ideas for advancing BLCA treatment in the future and improve patients' responsiveness to ICB treatments.

## Data Availability

All datasets generated in the present study can be found in online databases. The names of databases and accession number(s) are provided in the article. All the data can be obtained from the corresponding author upon reasonable request.
